# 

**Published:** 1891-03

**Authors:** 


CONTENTS:

PAGE

ORIGINAL ARTICLES:
- Hyposulphite of Soda. By E. H. Sheperd, V. S. .... 109
- The Germ of Texas Fever. By R. R. Dinwidde, Veterinarian .... 115
- Age of the Horse, Ox, Dog, and Other Domesticated Animals. By R. S. Huidekoper, M. D., Veterinarian .... 118
- Animal Intelligence. By H. Vulliamy .... 121
- Pennsylvania State Veterinary Medical Association .... 125
- Leukaemia. By Dr. George G. Vandeveer .... 126
- Influenza in Horses. By Dr. George Fleming .... 129
- Extent of Reason in the Lower Animals .... 137
- Recent Patents .... 141
- Necrology .... 145
- State of Wisconsin .... 146

SOCIETY PROCEEDINGS:
- California State Veterinary Association .... 147
- New Jersey State Veterinary Society .... 149
- United States Veterinary Medical Association .... 150
- Ontario Veterinary Medical Association .... 150
- New York State Veterinary Society .... 151

REVIEW DEPARTMENT .... 155

